# Finding What Is Inaccessible: Antimicrobial Resistance Language Use among the One Health Domains

**DOI:** 10.3390/antibiotics10040385

**Published:** 2021-04-03

**Authors:** Lauren L. Wind, Jonathan S. Briganti, Anne M. Brown, Timothy P. Neher, Meghan F. Davis, Lisa M. Durso, Tanner Spicer, Stephanie Lansing

**Affiliations:** 1 Department of Biological Systems Engineering, Virginia Tech, Blacksburg, VA 24060, USA; 2 University Libraries, Virginia Tech, Blacksburg, VA 24060, USA; jonbrig@vt.edu (J.S.B.); ambrown7@vt.edu (A.M.B.); tanner9@vt.edu (T.S.); 3 Department of Agricultural and Biosystems Engineering, Iowa State University, Ames, IA 50011, USA; tpneher@iastate.edu; 4 Johns Hopkins Bloomberg School of Public Health, Baltimore, MD 21205, USA; mdavis65@jhu.edu; 5 USDA-ARS, Lincoln, NE 68583, USA; lisa.durso@usda.gov; 6 Department of Environmental Science and Technology, University of Maryland, College Park, MD 20742, USA; slansing@umd.edu

**Keywords:** one health, antimicrobial resistance, antibiotic resistance, human, animal, environment, text data mining, natural language processing, common language, AMR, AR

## Abstract

The success of a One Health approach to combating antimicrobial resistance (AMR) requires effective data sharing across the three One Health domains (human, animal, and environment). To investigate if there are differences in language use across the One Health domains, we examined the peer-reviewed literature using a combination of text data mining and natural language processing techniques on 20,000 open-access articles related to AMR and One Health. Evaluating AMR key term frequency from the European PubMed Collection published between 1990 and 2019 showed distinct AMR language usage within each domain and incongruent language usage across domains, with significant differences in key term usage frequencies when articles were grouped by the One Health sub-specialties (2-way ANOVA; *p* < 0.001). Over the 29-year period, “antibiotic resistance” and “AR” were used 18 times more than “antimicrobial resistance” and “AMR”. The discord of language use across One Health potentially weakens the effectiveness of interdisciplinary research by creating accessibility issues for researchers using search engines. This research was the first to quantify this disparate language use within One Health, which inhibits collaboration and crosstalk between domains. We suggest the following for authors publishing AMR-related research within the One Health context: (1) increase title/abstract searchability by including both antimicrobial and antibiotic resistance related search terms; (2) include “One Health” in the title/abstract; and (3) prioritize open-access publication.

## 1. Introduction

Recently, global interdisciplinary efforts to treat infectious diseases and prolong the efficacy of antimicrobial drugs are starting to be conceptualized using the One Health model [1,2]; the collaborative and transdisciplinary approach to connect human, animal, and plant health to their environmental health [3]. Success of these efforts is often dependent on effective communication across the One Health disciplines at local, regional, national, and global scales; and among scientists, policy makers, and the public. Historically, human and animal health have been viewed and treated as two distinct disciplines [4], typically segregated among practitioners, policy makers, and academics despite acknowledgment of their linkages through ‘One Medicine’ [4]. However, the need to address emerging zoonotic diseases with an interdisciplinary approach has become increasingly evident. An interdisciplinary approach is especially needed and called for with the emergence and reemergence of pathogens and drug-resistant pathogens, such as Escherichia coli O157:H7, avian flu H5N1, swine flu H1N1 [5], and more recently with SARS–CoV–2 (COVID–19) [6]. Now called One Health, the concept integrates human, animal, and environmental health, including both natural sciences and human dimensions, in a single holistic approach to address public health concerns, including antimicrobial resistance (AMR) [2]. 

A One Health approach is key to addressing the complex, overlapping, and embedded subsets of problems associated with AMR [7]. The overwhelming success of antimicrobial drugs in treating infectious diseases during the last century contributed to their wide adoption in both human and veterinary medicine [8], and plant agriculture [9]. The same trend has been observed for anti-viral treatments, such as those used to treat AIDS, as well as antifungal and antiparasitic drugs [10,11,12]. The biological phenomenon of resistance was noted almost immediately following the discovery of antibiotics [13], and it is well established that the use of antimicrobial drugs, even prudent use, selects for microbial resistance to the drugs [14,15,16]. Complicating efforts to control drug resistant pathogenic microorganisms is the fact that the resistance mechanisms can also be found in non-pathogenic bacteria and pristine environments [7,17], and the genes coding for AMR often reside on mobile genetic elements with the potential to be shared [18]. The environment then becomes a source and a sink of antibiotic resistant bacteria (ARB) and antibiotic resistance genes (ARGs) that can have serious consequences for human and animal health.

As with any multi-disciplinary approach, the individual disciplines use similar vocabularies, but each may have different preferred terms or ascribe the same words with different meanings or connotations [19], leading to potential miscommunication and barriers to collaborative success [20]. For example, using the word “environment” in human medicine may be directly related to the operating room to which a patient is exposed [21]. Within environmental health disciplines, the word “environment” corresponds to the physical, chemical, and biological external factors that may impact behavior and overall health [22], while in environmental science disciplines, “environment” refers to components of nature that support life, including soil, water, and air [22]. Specific to AMR research, some disciplines within the One Health approach use “antimicrobial resistance,” whereas others use “antibiotic resistance.” Researchers may or may not state the distinctions between these two terms, which challenges interdisciplinary communication and collaboration.

One Health bridges a widespread cohort of disciplines, which include but are not limited to environmental health, ecology, veterinary medicine, public health, human medicine, microbiology, and health economics [23]. There is a general consensus that the key terms and practices used in each of the One Health disciplines will inherently be different [24]. A One Health evaluation was conducted at the University of Copenhagen Research Centre for Control of Antibiotic Resistance (UC–CARE) to analyze how researchers from fourteen departments over four years could come together to produce new knowledge to reduce AMR [25]. Léger et al. [25] found that most interviewees had increased awareness and general understanding of AMR from a One Health lens. However, the challenges of information sharing, collaboration, and methods hindered the productivity of producing novel AMR findings. Additionally, the problems that arose from communication, and/or lack thereof, were linked to the overarching issue that there was no common scientific language across disciplines [25]. This evaluation highlights that language disparity among One Health domains needs to be quantified to identify language gaps. Understanding these disparities will aid in creating consistency and a common language within the One Health framework to increase AMR communication, support productive discussions, and enhance knowledge transfer across disciplines. As stated by Mendelson et al. [24], “Antibiotic Resistance has a language problem.”

The aim of the current study was to evaluate the variations in language usage among One Health researchers and their relevant disciplines (i.e., human, animal, environment). Specifically, the following questions were addressed: (1) Are there dissimilarities in key term frequency usage within the AMR published literature across the One health domains; (2) Does AMR associated language usage increase at similar rates across the One Health domains from 1990–2019, and (3) Are the AMR language usage frequency trends similar in open-access articles compared to non-open-access articles in the One Health domain? Quantifying the scale of language disparities among the One Health domains will improve future searchability and accessibility to create a more inclusive and collaborative understanding, while continuing to promote interdisciplinarity within AMR research. This quantification using neutral and replicable methods necessitated bringing together an interdisciplinary group of scholars across the One Health domains and experts in text data mining (TDM) and natural language processing (NLP). The team performed an open-access search of all One Health and AMR relevant available publications in European PubMed Central (Europe PMC), and analyzed the results across the three One Health domains (human, animal, environment). This study is novel in its application of natural text parsing and large data analytics to the One Health domains which, to our knowledge, has not been attempted before this effort. By including experts across the One Heath domain with data scientists from project conception through data analysis, this team was able to show the impact of language use dissimilarities on effective communication and publication access across the One Health disciplines.

## 2. Results and Discussion

The TDM analysis of open access AMR publications confirmed that language continues to be a significant barrier to communication across the One Health domains. We found that when writing about antibiotic resistance, each of the four term bins (Human, Animal, Environment, and the combined bin of One Health) had its own common language (i.e., theme) that did not overlap with the other bins. Additionally, the majority of articles recovered in our search used the term “antibiotic resistance” instead of “antimicrobial resistance,” regardless of the domain. We also identified temporal trends in language and acronym use from our investigation. The next sections summarize key findings in each of these areas and describe factors that may be important to consider for future research and language harmonization efforts within the One Health domains.

### 2.1. Language Use across the One Health Domains

**Figure 1:**
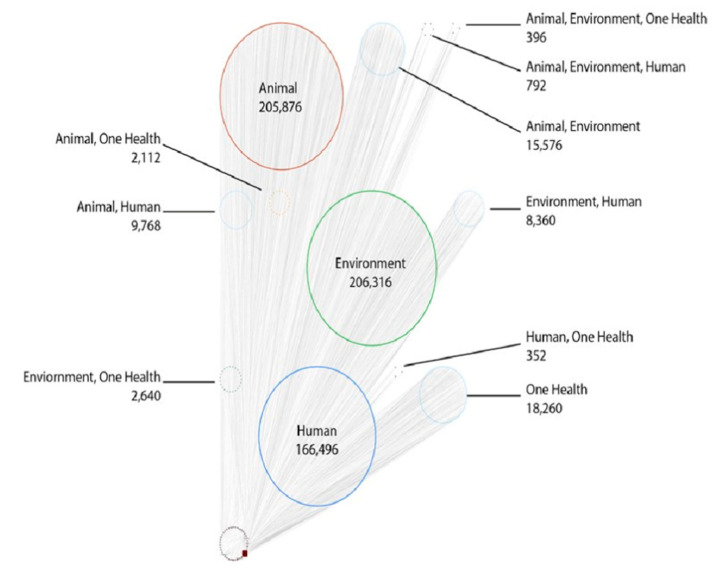
Network of the similar and/or dissimilar language usage among One Health groups. The Group Attributes Network Layout groups articles into bins and places each bin on the graph in relation to how similar the bins are to one another. Each bin represents different combinations of search terms found in the articles’ titles and abstracts. For instance, the Human group are articles that exclusively used human-binned terms in their title or abstract. Larger groups (and group counts) indicate the number of articles within that group. Bins plotted closer together share similar patterns of term frequency and usage within the articles’ body. The number within each circle indicate how many occurrences of those terms occurred in the articles (i.e., the terms in the human term bin occurred 166,496 times in the articles that exclusively used human terms in their title and abstracts).

There is broad support among national and international groups to adopt an interdisciplinary and One Health approach in efforts to address AMR [26], with calls for a review of AMR terminology across disciplines to facilitate a productive and coordinated global response [24]. Using TDM, we analyzed key term frequency within 20,000 representative AMR articles to determine consistent, and at times inconsistent, language use within articles categorized into four independent term bins (Human, Animal, Environmental, and One Health bins). As language use is key to interdisciplinary group dynamics, terminological imprecision can result in dissimilar vocabulary that presents a barrier to moving to the shared cognition required for strategic interdisciplinary problem solving [19,25,27,28,29,30], particularly as it relates to AMR [24]. We identified that when using a consistent search term string, the key term usage deviated amongst the individual domains, with search term clusters shown within each bin (Figure 1). Term frequency and usage becomes increasingly less consistent with articles containing terms from multiple domains. Articles that used human and animal terms in the title and abstract used the term ‘human’ 2x more and ‘ecosystem’ 11x less on average within the article body than articles with human and environment title and abstract terms. ContentMine identified search terms that were specific to each of the four bins. However, there was no distinct overlap in the language used across the One health domains. The limited overlap observed in the title and abstract key terms highlights the lack of shared language, and potentially shared cognition, among AMR researchers in different One Health domains (Figure 1).

These results highlight the information gap among fields. Open-access, peer-reviewed AMR research content largely focuses on a singular domain concept, with primarily superficial links between the disciplines, which others have noted [20,31]. The new findings from this current study on language use frequency also support this claim. It should be noted that this method of text processing is not encompassing of the totality of how NLP can be applied. This foundational work determined language trends and the methods of how new analyses types, such as NLP, can be applied to the One Health field. In order to overcome the language use barriers, we recommend the following: (1) researchers from specialized disciplines be trained to search for multiple search terms encompassing each One Health domain in title/abstract literature searches, and (2) researchers be trained to include multiple search terms encompassing all of the One Health domains in their title/abstracts when writing AMR focused papers.

### 2.2. Trends in “Antimicrobial Resistance” Language Use over Time

**Figure 2:**
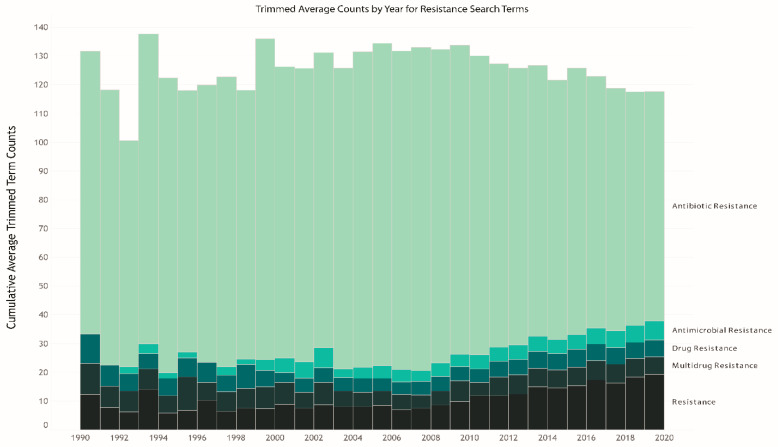
The average trimmed term counts of resistance-related search terms each year used per article. Trimmed search term counts, top and bottom 10%, were removed as assumed outliers.

The raw number of peer-reviewed One Health-related AMR articles indexed in Europe PMC increased almost five-fold from 1990 (n = 33,362 articles) to 2019 (n = 165,516). Each One Health discipline or sub-discipline had different underlying assumptions and understandings of the terms “antibiotic resistance” and “antimicrobial resistance.” In the representative 20,000 articles used for TDM analysis, the search term group for antibiotic resistance, consisting of “antibiotic resistance” and the known associated acronym (“AR”) was used on average 18 times more often than the antimicrobial resistance term group consisting of “antimicrobial resistance” and its known acronym (“AMR”) (Figure 2). However, since 2009, there has been a marginal, yet discernable, increase in other resistance related terms being used (i.e., multidrug resistance, one health, antimicrobial resistance) and a substantial decrease (26%) in the frequency of the antibiotic resistance term group (Figure 2). This was unexpected, but the decrease in frequency use of “antibiotic resistance” and “AR” key terms suggests that authors may be narrowing their scope of AMR research to identify more closely with specific research objectives (e.g., limited to a single gene) rather than a broader scope discussing “antibiotic resistance” in general. Additionally, Krockow [32] argued that a new name is needed for AMR due to the inconsistent use of AMR in the literature, difficulty in pronunciation, and unclear meaning to lay audiences, but a new name was not suggested. Our analysis shows that the literature is already starting to move away from the more general AMR term to more specific terms within sub-fields in the One Health context.

### 2.3. Trends in Acronym Use over Time

We identified that acronyms (e.g., AMR) are used commonly in text rather than in the title/abstract title/abstract among the included articles. Researchers within the AMR One Health field have worked towards creating a common glossary of terms and acronyms related to “antibiotic resistance” [33,34,35]; however, the study results show there is still a lack of consensus on the meaning of many terms and disagreement, or inconsistency, in which term or acronym should be used when publishing in a One Health context. In addition to “AMR” and “AR”, “MDR” is widely used for “multidrug resistance.” We found fluctuations in the popularity of “AMR” vs. “MDR” vs. “AR” over time (Figure 2). The median frequency of “AR,” “AMR,” and “MDR” after TDM averaged 230.0, 9.5, and 13.5 average term counts for each article, respectively (Table S1). This supports the concept that the acronym usage remains consistent overtime (i.e., researchers are using “antibiotic resistance” and “AR”), but that subfields within each domain have seen increased popularity over time for other related resistance terms. Creating a common language will aid to bridge communication gaps between the domains. Potential solutions to language barriers include harmonization to a single term/abbreviation and/or training researchers to include both “AR” and “AMR” in title/abstract to increase searchability across domains.

### 2.4. Trends in Language Use among the One Health Domains

**Figure 3:**
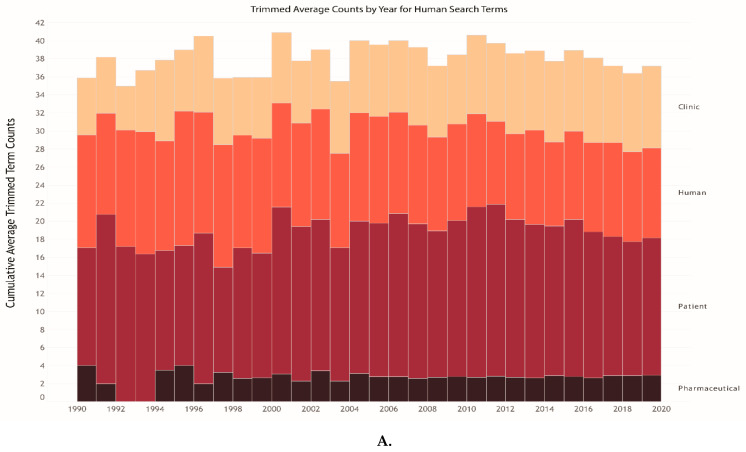

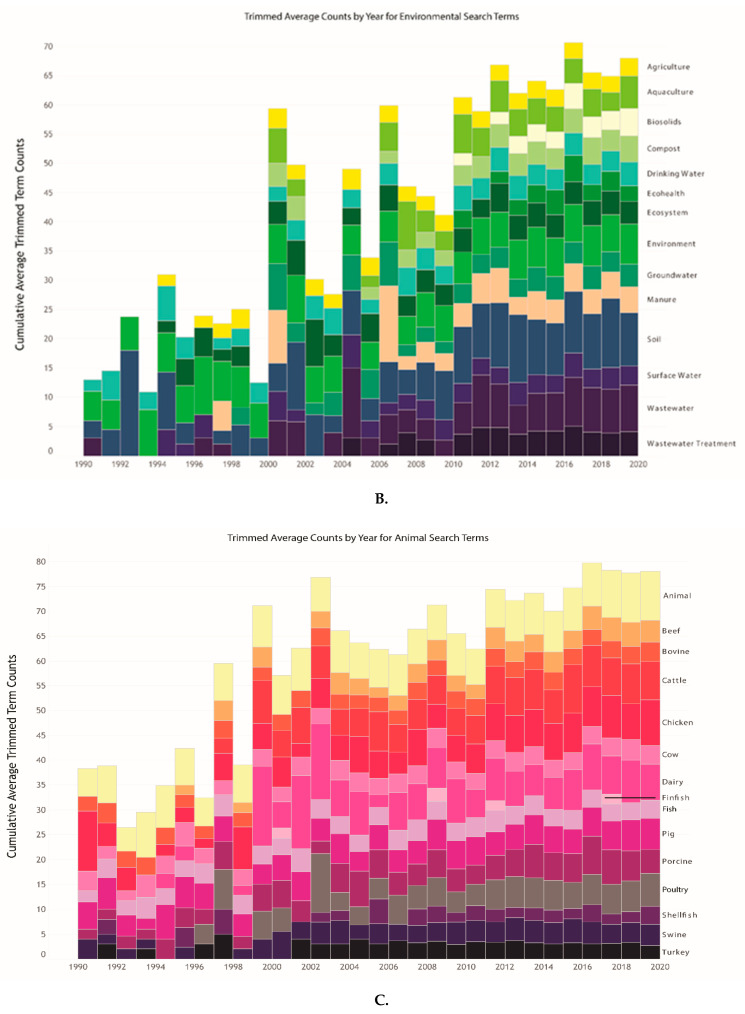

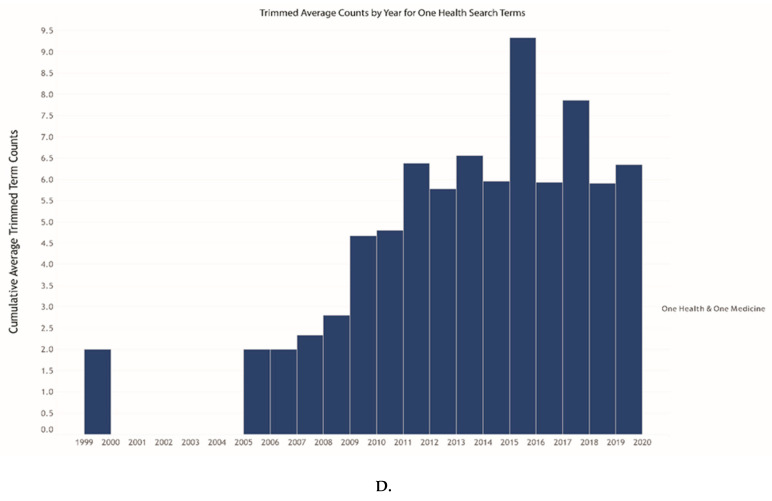
The cumulative average trimmed (top and bottom 10 percentile omitted) term ounts from 1990 to 2019, binned by the One Health domains. Returned articles that contained search terms related to antimicrobial resistance in title and/or abstract are shown based on bins: (**A**) Human-associated, (**B**) Environmental-associated, (**C**) Animal-associated, and (**D**) One Health associated.

Over the twenty–nine year span studied in this work, there was an upward trend in the usage frequency of the term “One Health,” indicating that more articles include the One Health concept in their studies or are using “One Health” within their AMR related research articles. Consistent with prior work [36], our analysis further indicates that Human-associated key terms were two times more likely to be used than environmental and animal associated key terms in articles addressing AMR (Figure 3A–D). In 2013, the Animal and Environmental-associated search terms began to increase, while the human-associated search term frequency began to decrease in all articles. It is unsurprising that the human associated search terms were more frequently being used at the beginning of this study period due to the larger focus on AMR research in human and clinical studies in the 1990s. Using TDM, we identified that the Animal, Environment, and One Health binned associated search terms began developing in the late 2000s and continued to increase in frequency over time. This trend correlates with broader adoption of the One Health concept and an increase in funding for animal and environmental AMR research, which began in the late 2000s and is ongoing with the creation of national and international One Health funding programs and initiatives, such as the Joint Programming Initiative on Antimicrobial Resistance, the US Center for Disease Control (CDC) AMR Fund, and the US Department of Agriculture’s National Institute of Food and Agriculture (USDA-NIFA) AMR funding initiative [37,38,39]. Although Human-associated key terms were used more frequently in human grouped articles over the 29-year analysis period, there was no significant difference between the average usage frequency of key terms between Human, Animal, Environmental, and One Health binned articles (2-way ANOVA; p = 0.482; Table S2). However, when all overlapping domain (i.e., animal-human, human-environment, environment-animal, animal-environment, etc.) articles were considered, there were significant differences between key term usage frequency and binned domain (2-way ANOVA; p < 0.001; Table S2). This suggests that once articles are grouped by sub-specialties within each One Health domain, differences in common language use throughout the entire article can be identified. 

**Figure 4:**
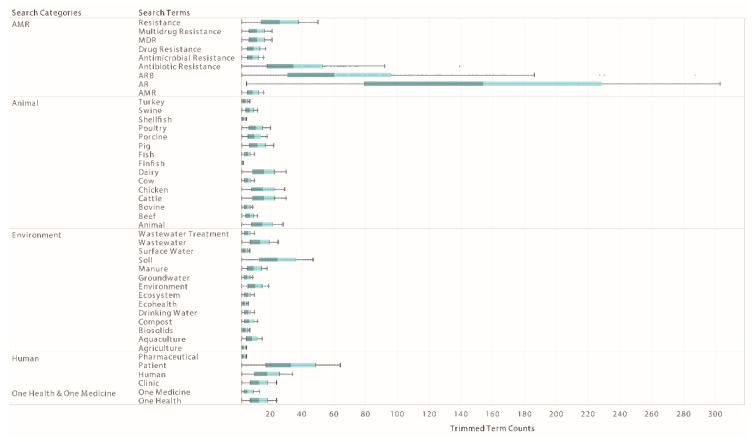
Box and whiskers of the top and bottom term counts in each of the term bins.

For each One Health domain, the most and least frequently used search terms from 1990–2019 ranged from 6.9–3.9 average trimmed counts within AMR related articles (Figure 4, Table S1). For Human binned articles, “patient” was the most frequently used (16.7 trimmed counts) and “pharmaceutical” was the least frequently used (2.8 trimmed counts) search terms. For Animal binned articles, “dairy” was the most frequently used (7.7 trimmed counts) and “finfish” was the least frequently used (2.3 trimmed counts) search terms. Interestingly, within the Animal binned articles, “dairy,” “cattle,” and “chicken” were the top three search terms and had similar frequencies (7.7, 7.6, 7.3 trimmed counts, respectively). For Environmental binned articles, “soil” was the most frequently used (9.4 trimmed counts) and “agriculture” was the least frequently used (2.8 trimmed counts) search terms. A notable similarity among the three bins is that the most frequently used search term was associated with the physical environment that each bin influences, suggesting the journal articles on AMR research continue to direct their discussions only on the physical environments in their associated domain and fail to widen the discussion to the One Health context. The term environment is a known rich point that has different meanings among domains [20], which is supported by our word frequency analysis results. Future NLP analyses contextualizing how the word environment is used in One Health domains could elucidate these distinctions and patterns. 

### 2.5. Accessibility of AMR Articles, Publication Preferences, and Potential Biases among the One Health Domains 

#### 2.5.1. Non-Open/Restricted Access vs. Open Access Journals

A TDM analysis relies on unrestricted access to literature. To explore potential biases among the used methods that only include open access literature, we performed a case study of a non-open access journal in the Animal domain, as the Animal domain had a higher proportion of non-open access journals related to AMR. The Journal of Dairy Science (JDS), a non-open access journal, was selected for the case study, with language usage in articles from JDS compared to the studied set of open-access articles. As non-open access journals are available only to subscribers, the audience has a more focused expertise, likely to convey a more niche perspective than open-access audiences. The comparison between resistance (i.e., “AMR,” “AR,” and “MDR”) search terms in open-access journals and JDS showed that the language usage patterns were similar overall. The term “antibiotic resistance” was used more frequently over time in open-access journals compared in JDS, however, the term “antibiotic resistance” was the most frequently used AMR term in the analyzed JDS articles. Interestingly, the search term “resistance” increased in usage in both datasets, but was proportionately used more frequently in JDS, suggesting that other terms relating to resistance (i.e., viral, pesticide, etc.) are populating the field recently (Figure S1). “One Health” or “multidrug resistant” were not used in any of the JDS subset articles, in contrast to the open-access articles. This result may be indicative of JDS authors obtaining funding from sources that do not require open access publications, and/or the more subject-specific focus of researchers publishing in JDS compared to open-access sources. Furthermore, while “antimicrobial resistance” usage was rising in the open-access journals, it was steady over time (1990–2019) in JDS. The range of One Health associated search terms used in JDS was expected to be smaller compared to the large dataset of open-access journals due to the niche JDS audience. However, the result that the term “One Health” was not found in journal articles from JDS may suggest that One Health articles are more likely to be published in journals specifically geared towards the One Health concept rather than in animal-specific journals. While not all search terms were present within the JDS sample, the represented search terms maintained a similar average term count throughout the time span studied, suggesting that the omission of non-open/restricted access journals in this study potentially biases towards the overuse of One-health related terms, but not resistance search terms.

#### 2.5.2. Publication Preferences

The 1.2 million articles returned on Europe PMC were retrieved from 4239 unique journals (Tables S3–S7). The inherent diversity and potential bias in choosing where to publish highlights the importance of using appropriate search terms and key words to find relevant articles. For example, a scientist studying the effects of antibiotic usage in dairy cows may never find a useful, relevant article if they only searched within animal science and not One Health or environment-associated journals. PLOS ONE, Frontiers in Microbiology, and One Health were the open-access journals that returned the most One Health binned articles in our TDM analysis (Table S3). In another example, there were not any One Health binned articles (i.e., one-health, one-medicine) from the British Journal of Cancer, which highlights that articles that specifically use “One Health” or “One Medicine” in their title/abstract are not publishing in the same journals as the Human, Animal, and Environmental subdisciplines. This finding mirrors what was identified with the JDS case study. As the One Health domains gradually become more communicative and use common language, the focus on where to publish to reach the most researchers in Human, Animal, and Environmental disciplines will become more judicious. To increase communication and common language usage within One Health a preference should be given to publishing open access. 

#### 2.5.3. Potential Biases among the One Health Domains

Providing data lake application programming interfaces (APIs) and NLP processing tools with a specific set of search terms inherently introduces bias into the results. These terms were iteratively collated by the domain experts and referenced against ontologies to provide potential synonyms. Without providing these tools with an initial direction, these results would culminate in discovering the most commonly used word, overall, in the EuroPMC repositories. This search term bias is unavoidable at project conception, but can be mitigated by future programmatically driven analysis to identify alternative principal agents that may be responsible for the trends seen. While we cannot state that all findings are concisely linked to the terms we have identified, there is confidence that these results do indicate that term usage discrepancies exist, and that the dissimilarities must first be identified consistently before domain-level changes can be made.

Additional biases can be shown by authors or search term users through both Journal choice for publication, and more importantly, when searching for relevant AMR literature using search terms in title/abstract. The search strings used for the four binned groups in this study came from multiple brainstorming sessions with the authors, librarians, and a group of AMR-focused researchers, veterinarians, economists, and extension specialists from a USDA-NIFA funded workshop (detailed at https://osf.io/g7amj/, accessed on 1 April 2021). After using TDM to gather the count frequency of the search terms from the queries, the top 100 words from each domain were analyzed. The NLP analysis identified several terms that were not part of our search queries (Table S8). Among these, the word “cell” occurred most frequently in all three domains (Human, Animal, and Environment), with “study,” “gene,” and “protein” also found in all three domains. The human and animal group shared four additional terms (“patient,” “cancer,” “human,” and “expression”), possibly due to the conceptual links and overall health goals of both human and veterinary medicine. In contrast, the human and environment group only shared one additional term (“treatment”). We suspect that the term refers to caring for a patient in the Human domain and processing wastewater in the Environmental domain, another example of how the same word conveys different meanings across One Health teams. The Human and Animal groups each had one word in the top ten list that was unique to their group (“disease” and “level,”, respectively). The Environment group had four terms that were unique, highlighting fundamentally different perspectives and subject matter of the environmental pillar compared to the other two groups. Interestingly, the search term “resistance” was within the top ten search terms found only in the Animal and Environmental groups. The top ten words returned among all the binned articles included words specific to molecular research levels (i.e., cell, gene, protein, expression, and cancer) (Table S8).

Within the last decade, AMR articles have shifted to focus on specific microbial, genetic, ecological, public health, and disease mechanisms as critical research questions. The TDM analysis illuminated this shift and can be used to help researchers and policy makers within the One Health domains better understand which AMR related research areas are growing and which areas of growth are needed within each domain and across domains. One hypothesis for the unique and rather clumped molecular and medically related terms being more frequently used in all the analyzed articles may be related to funding sources within each domain. For example, the National Institute of Health (NIH) established Public Access Policy in 2008 that required all research funded by NIH to be published in an open-access format [40]. However, most Animal and Environmental AMR funding sources (i.e., US USDA, and US CDC) do not require research to be published open access. Promotion of open-access publishing among the animal and environmental domains could partially address this gap.

#### 2.5.4. Implications for Accessing the Literature

Given the differences in language use among the domains and over time, barriers likely exist for researchers to access publications from different disciplines. Differences exist in how major search engines identify publications based on keywords and/or controlled language [41]. For example, in PubMed, a search for antibiotic resistance returns the following search: “drug resistance, microbial” [MeSH Terms] OR antibiotic resistance [Text Word]. In contrast, a search for antimicrobial resistance returns a different search string: “anti-infective agents” [All Fields] OR “anti-infective agents” [MeSH Terms] OR antimicrobial [Text Word] AND resistance [All Fields]. This work identifies a need for researchers to consider using keywords and controlled language that may be outside their discipline, with the understanding that different assumptions and search algorithms of the various databases (Europe PMC, PubMed, Agricola, Scopus, Web of Science, etc.) will not capture all relevant publications across the One Health domains when language use is domain specific. This lack of a common language used in AMR-related articles limits results being produced from search that span across disciplines, which limits engaging in interdisciplinary team science due to the lack of common language usage across the One Health domain.

### 2.6. Using Text Data Mining to Predict Patterns of Historical and Future Events

TDM was valuable in detecting a decreasing trend in use of “antibiotic resistance” following 2009, which highlights the importance of understanding potential associated historical events that may have substantial effects on language usage in the One Health arena. In 2009, the Pandemic A(H1N1) outbreak occurred, claiming the lives of 123,000–395,600 people worldwide [42]. While this could be coincidental, it is also possible that the resources being used to study AMR within the One Health domains shifted to focus on the influenza outbreak, decreasing antibiotic-resistance related research and ultimately publications in 2009 and beyond [43,44]. The TDM analysis provided critical insight into a potential shift in research within the One Health domains after the 2009 HIN1 pandemic, which resulted in a 26% decrease in resistance related terms in 2010. At the time of publication of this work, the outbreak of the novel COVID-19 coronavirus is ongoing. It will be interesting to consider whether this trend may be seen again in coming years as research shifts focus to antiviral associations, despite concerns with breakdowns in antimicrobial stewardship given new reliance on telemedicine [45,46].

The relationships between AMR common terms and common misconceptions may be another communication issue for One Health moving forward. For example, antimicrobial soaps and sanitizers have been marketed for COVID-19 protection [47], and AMR is now being linked to COVID-19 [48,49]. This COVID-19 era highlights the importance of communication and having a common language to inform all stakeholders (i.e., essential workers, health care workers, school teachers etc.) of the current situation. Although AMR research is 20-30 years ahead of the COVID-19 pandemic, there is not a consensus surrounding AMR language within the One Health domains. Similar to the AMR research conducted here, TDM and NLP techniques can be used in the future to understand the beginning trends of COVID-19 research and how common language, or inconsistent language, was used during the COVID-19 pandemic. Currently, there is not enough academic literature to conduct this analysis, but in time, this type of study can be used to identify and coalesce a common language for COVID-19 related research without having to wait a quarter of a century to do so.

## 3. Materials and Methods 

### 3.1. Open Access Data Collection

ContentMine’s GetPapers [50] was utilized to create a text data lake (centralized repository) of relevant academic articles based on our search terms (Table 1) for each One Health domain. GetPapers is an open-source text mining tool, ran on NodeJS, designed to identify and retrieve open-access, peer-reviewed, full-text articles from Europe PMC [51]. GetPapers can pull the full text and metadata from Europe PMC, IEEE, ArXic, and Crossref via their respective APIs (application programming interface). These APIs allow for large scale recursive search and retrieval of articles without the need to manually do so via the website. This enabled automated replicable data collection to mine the large numbers of articles necessary. By using GetPapers, our search queries had the same functionality and parameters as searching those repositories directly with the added benefit of automating the large-scale collection. An example of the GetPapers Europe PMC query syntax can be found in the Supporting Information (Figure S2). Multiple terms related to the same topic, such as “multi-drug resistance” and “antibiotic resistance” were pulled/retrieved if the domain experts felt that the terms are used interchangeably or in different frequencies within the term bins. The intention of this data pull was to pull as many related articles to these search terms, and redundant terms increased the potential search sample. Each search domain category (Human, Animal, Environment, One Health) is classified as a term bin. The search was restricted to articles written in English. Full-text articles were returned as both PDF and JavaScript Object Notation (JSON)-formatted files that were binned into groups (i.e., bins), initially, based on the One Health search domains. NodeJS was used to run the ContentMine library. Workflow of the data collection, processing, and visualization is detailed in Figure S3.

Initially, One Health specific key terms (i.e., one health, one medicine) were included in each binned search query. However, that resulted in the binned queries occasionally returning articles that only contained One Health terms in the title or abstract, and not being related to AMR. These hits artificially raised the total counts per bin without providing data that informed the experimental questions related to AMR language use across the One Health domains. To enhance relevant sample size and text representation in all domains, One Health as a search term was added as a fourth term bin (i.e., domain), and articles were further processed downstream to confirm utilization of One Health terms and utilization with respect to AMR as an additional group (Table 1).

In total, 1.2 million articles were returned from the Europe PMC search queries for all articles by search term bins (Human, Animal, Environment, and One Health; n = 4) by title/abstract. To create a workable and representative sample size, up to 2000 articles were pulled from Europe PMC for each search term bin for every year from 2000–2019. Additionally, a data pull was done for each search term bin for the years 1990–1999, again with a 2000 article cap. As there were less articles in Europe PMC during that duration, one pull was sufficient to gather the majority of the articles. All pulled articles were used to create a data lake of up to 168,000 total articles with full text and metadata. It was not possible to search the terms for each bin without yearly constraints, as the EuroPMC API defaults to providing the most recent articles first. By specifying and running multiple queries per term bin, we were able to ensure that every year was represented, and no year was overly represented in the sample, as long as papers with those terms existed during that year. From this article data lake, 5000 randomly selected articles were pulled for each term bin to create a dataset of 20,000 articles for downstream TDM analysis. This created fixed, uniform sample sizes for each term bin that could then be analyzed.

### 3.2. Non-Open Access Data Collection

The Journal of Dairy Science (JDS) was used to descriptively compare the TDM results from open-access vs. non-open/restricted access articles within the animal domain using the same search queries as the ContentMine data mining process presented above (Table 1). As stated in Buyalskaya et al. [52], the silos that exist within journals that cater to the readership of a specific discipline may limit access to information for interdisciplinary researchers within the One Health context. JDS was chosen as an example discipline-specific journal, because the manual review of an initial search revealed that the animal domain more frequently had relevant AMR articles in non-open/restricted access journals compared to the other domains surveyed. In addition to its clear categorization within the animal domain of the One Health triad, the JDS data structure allowed for an automated search via EBSCOhost database using the same queries that were used to search Europe PMC via ContentMine. Unlike the millions of open-access articles returned from Europe PMC that were subset into 5,000 articles per topic bin from 1990–2019, the JDS search queries returned 689 Human, 1,853 Animal, 340 Environment, and 14 One Health binned articles. A sample (40%) of each topic bin was randomly downloaded for returned articles (saved as PDF) using EndNote, and the saved PDFs were used to compare the language and key term frequency use between open vs. non-open/restricted access articles.

### 3.3. Text Data Mining Processing

A total of 20,000 open-access articles from Europe PMC and 972 non-open/restricted access articles from Journal of Dairy Science were collected as PDFs in the four topic bins and further analyzed. All search queries, keyword binning, manuscript retrieval and processing methods, script files, and raw data can be found on our Open Science Framework (OSF) site (https://osf.io/g7amj/, accessed on 1 April 2021). While our search queries specified open access articles, which primarily have a CC BY license attached, we have not provided the individual text articles, as the exact copyright requirements for each document cannot be assumed. These search queries are specific and will pull a near identical set to the data we collected.

Natural Language Processing (NLP) trained libraries were used to extract data from published peer-reviewed articles. NLP, among other functions, creates structure out of unstructured text through analyzing, understanding, and deriving meaning from human written language [53]. Using NLP can give researchers insight into the meaning, purpose, sentiment, and more of natural (i.e., human-written) text information that was previously locked primarily behind individual or group manual interpretation. Downloaded PDFs were first converted to plain text files using the python package pdf2text. The NLP package NLTK (www.nltk.org) was then used to create consistency with letter case style, remove punctuation, tokenize, remove stop words, and lemmatize the text. Lemmatization, in NLP context, means to reduce alternate forms of a word, for example “am”, “are”, and “is” are all grouped together under the verb “be”, and “cars”, “car”, and “car’s” are all grouped together under the noun “car.” 

The clean data were then merged with metadata (i.e., DOI, article title, year published, journal, PMC ID) from the paired JSON files for each article, and a combination of Pandas, a popular python library with integrated data processing functions, data frames and NLTK processing were used to calculate the term counts per article. Terms that were comprised of two or more words (i.e., antibiotic resistance) were counted with text processed in bi-grams. The total number of words per article was used to provide a percentage of term counts related to each unique article. Percentages based on the total word count were used to standardize against differing article lengths. Data were trimmed to remove articles that comprised the bottom and top 10% of search term frequencies. This was done to further normalize the data and omit outliers (i.e., a 48-page review on antibiotic usage in agriculture and related public health implications that used the word “resistance” 409 times (PMC6017557, [54]) compared to the average and standard deviation term count (16 ± 28) across all articles analyzed.

### 3.4. Data and Statistical Analysis

Tableau Desktop (Tableau Software, Seattle, WA) was used to process and analyze the merged data after cleaning [55]. Two-Way Analysis of Variance (ANOVA) was used to determine the variance between the top 25 search key terms frequencies counted (Text S1), as well as the key term frequency usage among their associated topic bins. The correlation coefficient was used to determine similarity of total key term frequencies between the topic bins. Additionally, correlation coefficients between the top 25 key terms (Text S1) were used to determine the potential of terms co-existing within an article, regardless of the articles’ original topic bin. Interactive Tableau visualizations to explore all avenues of the dataset can be found at the publicly available collection within the OSF site (https://osf.io/g7amj/, accessed on 1 April 2021). Cytoscape software (www.cytoscape.org) was used to create a network graph [56]. This network graph plotted the frequency in which each article used any of the search key terms (Table 1) in their title or abstract and allowed visualization of potential overlap in key term use between the four One Health domains.

## 4. Conclusions

Using TDM methods, our study highlights what many researchers in the One Health domains already perceive with respect to AMR: we do not communicate well outside of our trained disciplines, and this is reflected in the peer-reviewed literature. This is in part due to the differences in our use of key search terms, where we publish, and how we identify interdisciplinary and transdisciplinary research articles. Moving forward, we suggest the following for authors publishing AMR-related research within the One Health context: (1) increase title/abstract searchability by including both antimicrobial and antibiotic related search terms; (2) include “One Health” in the title/abstract and keywords; and (3) prioritize publishing open-access. Additionally, we suggest that in order to bridge the gap between the One Health domains, researchers need to incorporate specific, and multiple, search terms when looking for relevant AMR research (i.e., include both antibiotic and antimicrobial resistance term groups). Table 1 from this study can be used as a guide for choosing relevant terms. Throughout this article, we specifically chose to use antimicrobial resistance as our key term to include bacterial, fungal, parasitic, and viral resistance representation, following the common language used by the World Health Organization (WHO) for AMR global action plan and the World Organization for Animal Health (OIE). While we chose to use AMR here, our study found that antibiotic resistance was the more frequently used key term, suggesting that the use of different derivatives of this term (i.e., antimicrobial, multidrug, etc.) may not be accessed by One Health researchers actively searching for antibiotic and not antimicrobial. The keywords used to collect the articles, and in turn the data used, were intentionally terms that are broadly used throughout the One Health domain. This study acts as a first milestone on this research, reaffirming the notion that disparate language use within One Health is prohibitory towards collaboration and crosstalk between domains. With that milestone, a crucial next step will be to replicate this research and data collection with a new list of terms specific to one domain or research area to isolate examples of this happening, and begin qualifying and quantifying the language discrepancies.

Using the data produced from the TDM analysis in this study, we propose future research analyzes that address connotations of the language found in these articles using NLP techniques, such as sentiment analysis, topic modeling, and summarization. Further, these methods, both search term frequency and context, can be incorporated when analyzing writing intended for a greater audience (i.e., not solely peer-reviewed articles). This study can be scaled to include consumer and extension databases, web pages, blogs, and trade magazines to understand how AMR is communicated to those outside academia. Our python scripts have been made open source and are readily adaptable to new search terms, articles, or domains, enabling future language studies by researchers seeking to understand how topics and terms differ across disciplinary lines. Additionally, the links to our interactive Tableau dashboards can be found on our OSF site (https://osf.io/g7amj/, accessed on 1 April 2021). The strength of NLP lies in the fact that it can be used to predict the direction of the field based on past research. We hope that once a common language is established within One Health for AMR research, NLP can be used to predict the gaps and next steps to holistically address AMR globally through team science that is truly interdisciplinary; this starts with understanding differences in language use and language context. 

## Figures and Tables

**Table 1:** Specific search key terms binned by One Health domains used to query and return articles from ContentMine containing the key terms in the title or abstract, with at least one word from the ‘AND’ row in the article’s title or abstract and not articles returned with words from the ‘NOT’ row.

Term Bin	Human	Animal	Environment	One Health
Search Terms	Human, patient, pharmaceutical, clinic ^1^	Animal, dairy, cow, beef, cattle, poultry, swine, chicken, pig, turkey, fish, porcine, bovine, finfish, shellfish	Ecosystem, ecohealth, environment, soil, agriculture, wastewater, drinking water, groundwater, surface water, compost, manure, biosolids, aquaculture, wastewater treatment	One health, one medicine
AND	antimicrobial resistance, antibiotic resistance, drug resistance, multi-drug resistance, resistance, AMR, ARB, AR, MDR			
NOT	Herbicide, pesticide, disease resistance			

^1^ all possible endings to the root of “clinic” were included.

## Data Availability

Data is contained within the article.

## Supplementary Materials

The following are available online at https://www.mdpi.com/article/10.3390/antibiotics10040385/s1, Figure S1: JDS resistance search term frequency over time, Figure S2: Example of search query used within bin terms in ContentMine, Figure S3: Workflow, Text S1: The reduced list of the top 25 search terms, Table S1: Summary statistics of all searched terms, Table S2: ANOVA results, Table S3: Top “One Health” domain journals, Table S4: Top 10 “Animal” domain journals, Table S5: Top 10 “Environment” domain journals, Table S6: Top “Human” domain journal, Table S7: Top 10 combined journals for domain groups, Table S8: Top 10 words not searched for in the four binned queries.
