# Supplementary material for: Finding What Is Inaccessible: Antimicrobial Resistance Language Use among the One Health Domains
Source: Antibiotics (Basel). 2021 Apr 3;10(4):385. doi: 10.3390/antibiotics10040385 (PMC8065768; doi:10.3390/antibiotics10040385)
Supplement: Supplementary file 1 [file antibiotics-10-00385-s001.pdf]

antibiotics

MDPI

Article

Finding What Is Inaccessible: Antimicrobial Resistance Language Use among the One Health Domains

Lauren L. Wind [1,*], Jonathan S. Briganti [2], Anne M. Brown [2], Timothy P. Neher [3], Meghan F. Davis [4], Lisa M. Durso [5] ,Tanner Spicer [2] and Stephanie Lansing [6]

1 Department of Biological Systems Engineering, Virginia Tech, Blacksburg, VA 24060, USA; wlauren@vt.edu
2 University Libraries, Virginia Tech, Blacksburg, VA 24060, USA; jonbrig@vt.edu (J.S.B.); ambrown7@vt.edu (A.M.B.); tanner9@vt.edu (T.M.)
3 Department of Agricultural and Biosystems Engineering, Iowa State University, Ames, IA 50011, USA; tpneher@iastate.edu
4 Johns Hopkins Bloomberg School of Public Health, Baltimore, MD 21205, USA; mdavis65@jhu.edu
5 USDA-ARS, Lincoln, NE 68583, USA; lisa.durso@usda.gov
6 Department of Environmental Science and Technology, University of Maryland, College Park, MD 20742, USA; slansing@umd.edu
* Correspondence: wlauren@vt.edu

Citation: Wind, L.L.; Briganti, J.S.; Brown, A.M.; Neher, T.P.; Davis, M.F.; Durso, L.M.; Lansing, S. Finding What Is Inaccessible: Antimicrobial Resistance Language Use among the One Health Domains. *Antibiotics* **2021**, *10*, 385. https://doi.org/10.3390/antibiotics10040385

Received: 1 March 2021
Accepted: 31 March 2021
Published: 3 April 2021

Publisher's Note: MDPI stays neutral with regard to jurisdictional claims in published maps and institutional affiliations.

Copyright: © 2021 by the authors. Licensee MDPI, Basel, Switzerland. This article is an open access article distributed under the terms and conditions of the Creative Commons Attribution (CC BY) license (http://creativecommons.org/licenses/by/4.0/).

Scheme S1. Text. The reduced list of the top 25 search terms used for the correlation tests included: agricultural, human, fish, finfish, ecosystem, environmental, ecohealth, drug resistant, drinking water, dairy, cow, compost, chicken, cattle, clinic, bovine, biosolids, beef, aquaculture, ARB, AR, antimicrobial resistance, antibiotic resistant, animal, and AMR.

Table S1. Summary Statistics of key terms from 1990 to 2019.

Search Terms	Avg. Term Counts	Std. dev. of Term Counts	Avg. Trimmed Term Counts	Std. dev. of Trimmed Term Counts
Agriculture	2.8	4.1	2.8	1.0
Amr	9.5	22.6	5.4	4.0
Animal	11.4	18.6	8.4	6.7
Antibiotic Resistance	6.9	9.7	9.0	10.5
Antimicrobial Resistance	6.5	8.8	5.7	3.7
Aquaculture	6.5	12.9	4.7	3.3
Ar	230.7	237.2	174.3	67.6
Arb	7.1	13.9	9.1	14.9
Beef	5.2	10.1	4.2	2.8
Biosolids	3.9	9.2	3.4	1.5
Bovine	4.8	11.8	3.3	1.9
Cattle	10.9	20.8	7.6	6.9
Chicken	10.3	19.3	7.3	6.8
Clinic	11.2	21.6	8.5	5.8
Compost	7.3	21.6	3.9	2.5
Cow	6.2	17.2	3.6	2.0
Dairy	11.3	23.6	7.7	7.0
Drinking Water	4.8	8.6	3.8	2.1
Drug Resistance	6.6	9.8	5.6	4.0
Ecohealth	4.0	7.7	3.2	1.2
Ecosystem	5.7	15.9	4.0	2.2
Environment	8.3	13.4	6.3	4.6
Finfish	2.5	2.1	2.3	0.5
Fish	6.0	16.7	3.7	2.1
Groundwater	4.7	10.2	3.8	2.0
Human	14.4	20.7	10.5	8.1
Manure	7.9	21.3	4.5	3.5
Mdr	13.5	21.6	7.4	5.3
Multidrug Resistance	3.9	5.9	5.2	4.1
One Health	11.2	26.3	6.9	5.9
One Medicine	2.0	3.6	3.9	2.7
Patient	24.4	71.7	16.7	16.2
Pharmaceutical	3.4	7.2	2.8	1.0
Pig	9.8	23.1	5.5	4.6
Porcine	6.7	12.1	5.6	4.3
Poultry	8.0	14.6	5.9	4.6
Resistance	18.3	29.6	13.0	12.5
Shellfish	4.2	11.7	2.8	1.0
Soil	17.6	43.5	9.4	10.1
Surface Water	3.9	7.0	3.4	1.6
Swine	6.1	14.7	4.2	2.6
Turkey	4.3	9.4	3.2	1.4
Wastewater	10.6	22.7	7.1	5.8
Wastewater Treatment	4.8	7.4	4.2	2.4

Table S2. One Health Top Journals articles were returned from Europe PMC search queries.

Journal	Count
'PLOS One'	1188
'Frontiers in Microbiology'	1012
'One Health (Amsterdam, Netherlands)'	880
'Frontiers in Veterinary Science'	748
'Frontiers in Public Health'	704
'BMC Public Health'	704
'Infection Ecology & Epidemiology'	660
'Malaria Journal'	484
'Veterinary Sciences'	440
'BMC Veterinary Research'	396

Table S3. Animal Top Journals articles were returned from Europe PMC search queries.

Journal	Count
'PLOS One'	23,760
'British Journal of Cancer'	7832
'Frontiers in Microbiology'	6820
'Environmental Health Perspectives'	5500
'The Journal of Cell Biology'	5412
'Emerging Infectious Diseases'	4356
'Scientific Reports'	4004
'The Journal of Experimental Medicine'	3388
'BMC Genomics'	2948
'The Journal of General Physiology'	2288

Table S4. Environment Top Journals articles were returned from Europe PMC search queries.

Journal	Count
'PLOS one'	24,420
'Frontiers in Microbiology'	8536
'Environmental Health Perspectives'	7260
'Scientific Reports'	5324
'Emerging Infectious Diseases'	4620
'The Journal of Experimental Medicine'	4092

'International Journal of Environmental Research and Public Health'	3828
'BMC Microbiology'	3652
'British Journal of Cancer'	3564
'The Journal of Cell Biology'	3520

Table S5. Human Top Journals articles were returned from Europe PMC search queries.

Journal	Count
'PLOS One'	16,984
'British Journal of Cancer'	12,980
'The Journal of Experimental Medicine'	4708
'Emerging Infectious Diseases'	3960
'Environmental Health Perspectives'	2816
'BMC Infectious Diseases'	2552
'Malaria Journal'	2244
'The Journal of Cell Biology'	2068
'BMC Cancer'	2024
'Critical Care (London, England)'	1804

Table S6. Overlapping Top-Ten Journals returned from Europe PMC search queries.

Journal	Human	Animal	Environment	One Health
British Journal of Cancer	X	X	X	
Emerging Infectious Diseases	X	X	X	
Environmental Health Perspectives	X	X	X	
Frontiers in Microbiology		X	X	X
Malaria Journal	X			X
PLOS One	X	X	X	X
Scientific Reports		X	X	
The Journal of Cell Biology	X	X	X	
The Journal of Experimental Medicine	X	X	X	

Table S7. Top 10 words in the One Health domain binned articles.

Top Terms	Human	Sum Term	Animal	Sum Term	Environment	Sum Term
**1**	cell	1,680,634	cell	229,814	cell	137,997
**2**	patient	1,290,369	gene	103,338	gene	103,938
**3**	study	892,768	study	89,882	study	85,022
**4**	cancer	726,779	protein	78,061	resistance	75,139
**5**	treatment	559,664	human	66,467	protein	60,005
**6**	gene	547,230	resistance	62,872	strain	53,207
**7**	human	458,294	expression	58,888	data	47,785
**8**	protein	446,148	cancer	51,251	plant	45,789
**9**	expression	427,157	patient	46,105	water	43,826
**10**	disease	424,081	level	44,845	treatment	42,533

Table S8. Top 10 words in the One Health domain binned articles.

Top Terms	Human	Sum Term	Animal	Sum Term	Environment	Sum Term
1	cell	1,680,634	cell	229,814	cell	137,997
2	patient	1,290,369	gene	103,338	gene	103,938
3	study	892,768	study	89,882	study	85,022
4	cancer	726,779	protein	78,061	resistance	75,139
5	treatment	559,664	human	66,467	protein	60,005
6	gene	547,230	resistance	62,872	strain	53,207
7	human	458,294	expression	58,888	data	47,785
8	protein	446,148	cancer	51,251	plant	45,789
9	expression	427,157	patient	46,105	water	43,826
10	disease	424,081	level	44,845	treatment	42,533

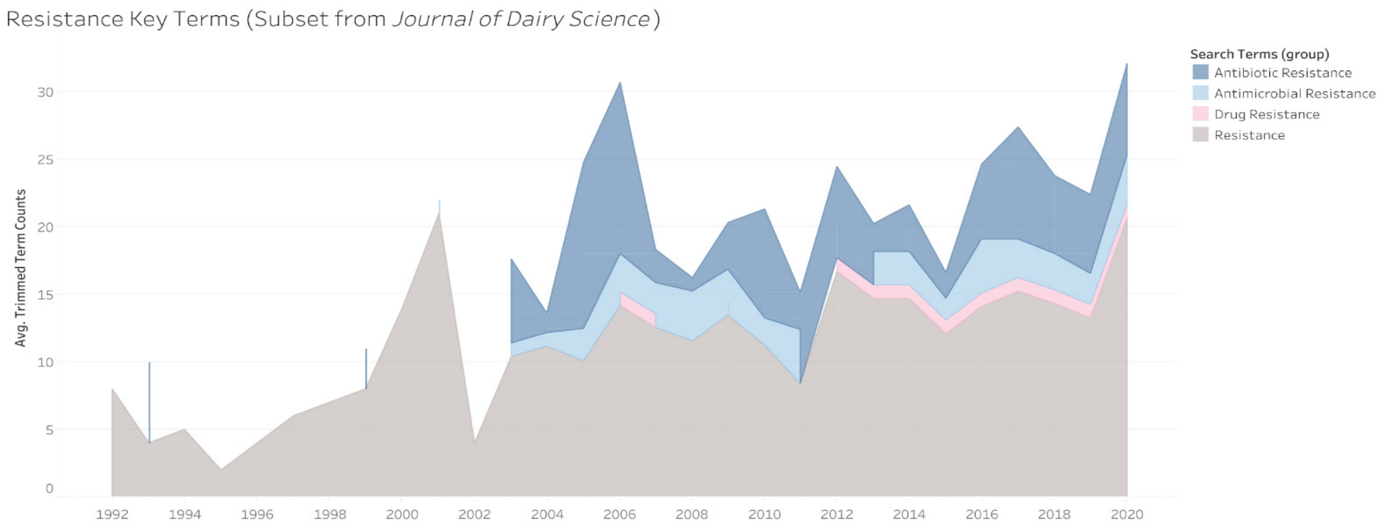

Figure S1. The average trimmed frequency counts of resistance-related search terms each year used per article in the Journal of Dairy Science. Trimmed search term counts, top and bottom 10%, were removed as assumed outliers.

```
node --max_old_space_size=4000 $(which getpapers) -q '(((TITLE:"human" OR
"patient" OR "pharmaceutical" OR "clinic*") AND (TITLE:"Antimicrobial
resistance" OR "antibiotic resistance" OR "drug resistance" OR "multi-drug
resistance" OR "resistance" OR "AMR" OR "ARB" OR "AR" OR "MDR") NOT
(TITLE:"herbicide" OR "pesticide" OR "disease resistance" OR "fungicide")) OR
((ABSTRACT:"human" OR "patient" OR "pharmaceutical" OR "clinic*") AND
(ABSTRACT:"Antimicrobial resistance" OR "antibiotic resistance" OR "drug
resistance" OR "multi-drug resistance" OR "resistance" OR "AMR" OR "ARB" OR
"AR" OR "MDR") NOT (ABSTRACT:"herbicide" OR "pesticide" OR "disease resistance"
OR "fungicide"))) AND (FIRST_PDate:[1990-01-01 TO 1990-12-31] AND HAS_PDF:y) '
-o HumanAmsContainer -p -f AmsContentMineHumanLog -k 2000
```

Figure S2. Example of search query used within bin terms in ContentMine.

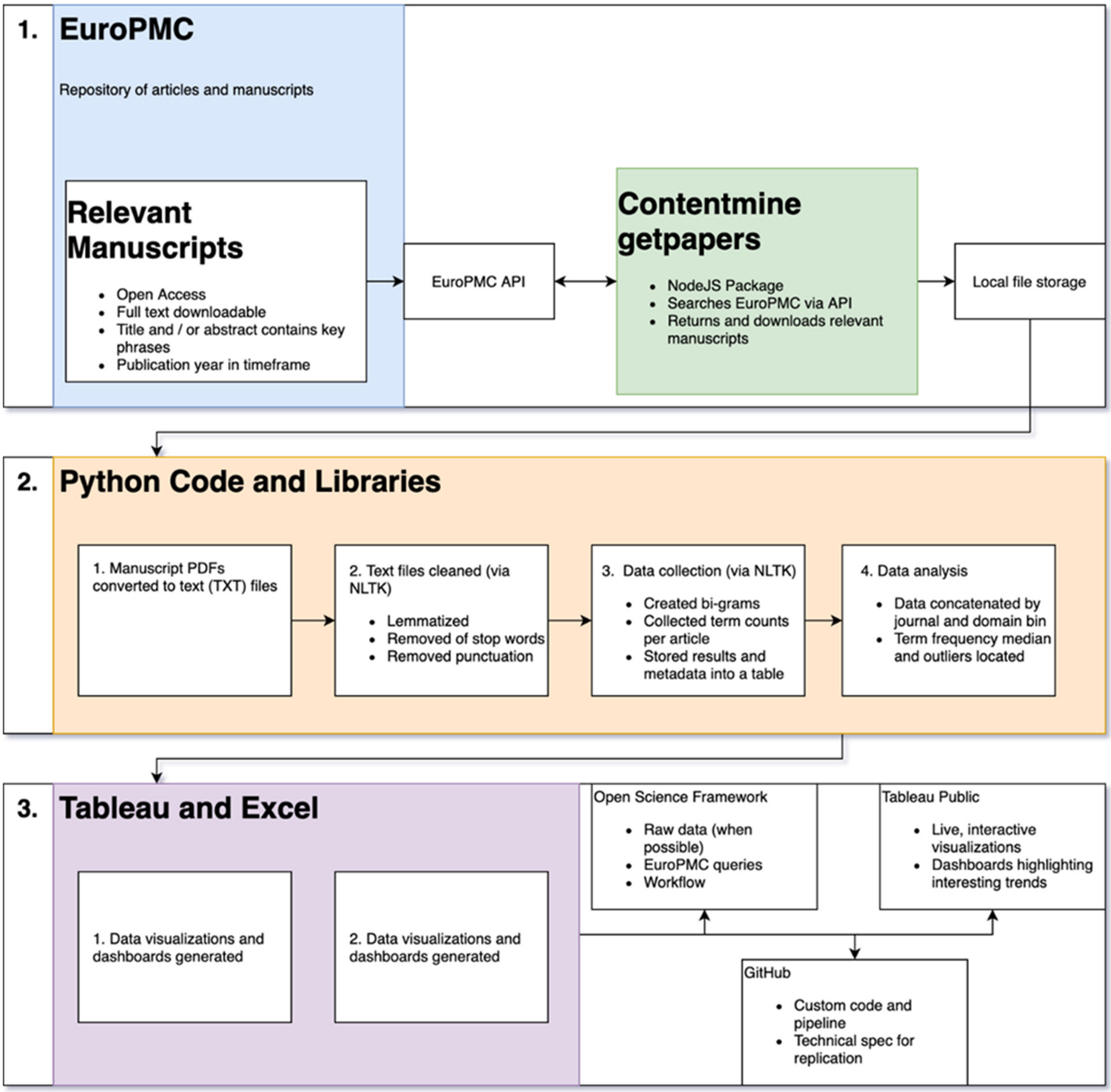

Figure S3. Workflow of the data collection, processing, and visualization details pertaining to this study.
